# The role of toxin–antitoxin systems in *Helicobacter pylori* adaptability

**DOI:** 10.1128/jb.00166-26

**Published:** 2026-08-12

**Authors:** Juan D. Romero-Carpio, Luis D. Chacón-Loría, Silvia Molina-Castro, Bradd Mendoza-Guido

**Affiliations:** 1 Instituto de Investigaciones en Salud (INISA), Universidad de Costa Rica 27915, San José, Costa Rica; 2 Departamento de Bioquímica, Escuela de Medicina, Universidad de Costa Rica 27915, San José, Costa Rica; 3 Centro de Investigación en Estructuras Microscópicas, Universidad de Costa Rica 27915, San José, Costa Rica; University of Virginia School of Medicine, Charlottesville, Virginia, USA

**Keywords:** gastric cancer, coccoid morphology, colonization, vapD, antibiotic tolerance

## Abstract

Toxin–antitoxin (TA) systems are genetic modules widely distributed across bacterial chromosomes and mobile genetic elements. Initially described as plasmid addiction systems that ensure vertical inheritance through post-segregational killing, they are now recognized as versatile regulators of bacterial adaptability. In *Helicobacter pylori*, a gastric pathogen infecting nearly half of the global population, multiple TA systems have been experimentally validated, including type I RNA–RNA modules and type II protein–protein pairs. These systems contribute to growth arrest, morphological transition from spiral to coccoid forms, biofilm formation, intracellular survival, and responses to environmental stressors, such as oxidative stress, metal availability, and antibiotic exposure. Type I AapA/IsoA modules have been linked to membrane targeting and dormancy induction, whereas certain Vap-like type II toxins act as ribonucleases and likely contribute to colonization and persistence during infection. Although hundreds of putative TA loci have been identified through bioinformatic analyses, only a small fraction has been functionally characterized, highlighting substantial knowledge gaps. This minireview summarizes experimentally validated TA systems in *H. pylori*, discusses predicted candidates, and examines their potential roles in pathogen adaptability, emphasizing their relevance to *H. pylori* persistence and pathogenicity.

## INTRODUCTION

Toxin-antitoxin (TA) systems are genetic modules typically composed of two closely linked genes: one encodes a stable toxin that disrupts essential cellular processes, while the other encodes a labile antitoxin that neutralizes the toxin’s activity. These genes are usually co-transcribed as an operon (1). TA systems were first discovered on conjugative plasmids, where they promote plasmid maintenance by linking cell survival to plasmid inheritance. When a cell loses the plasmid, the unstable antitoxin is rapidly degraded, allowing the toxin to act, thereby inhibiting growth or triggering cell death, an effect known as plasmid addiction (2). Nonetheless, it has been proposed that the role of TA systems in mobile genetic elements (MGEs) goes beyond plasmid addiction, contributing to their inherent selfish behavior (3).

Functionally, TA systems contribute to diverse physiological and ecological roles. In addition to plasmid maintenance, they mediate stress responses, persistence, antibiotic tolerance, defense against bacteriophages, programmed cell death, and bacterial dormancy. Some also serve as anti-addiction modules that neutralize plasmid-encoded counterparts (2). Currently, there are eight proposed types of TA systems according to the nature and mechanism of action of the antitoxins. Type II TA systems are probably the most abundant and diverse, consequently, being the best studied (2). In pathogenic bacteria, TA systems may enhance antibiotic tolerance, biofilm formation, genetic competence, and virulence, supporting infection and survival in hostile environments (4). It has also been suggested that a TA module is the origin of inter-bacterial and -kingdom effectors present in *Bartonella* species (5), highlighting their potential role in pathogenic adaptation.

With the advent of genome sequencing, homologous TA modules were also identified in bacterial chromosomes. However, these chromosomal systems are often associated with MGEs, including integrative and conjugative elements (ICEs), transposons, prophages, and integrons, or may exist independently as genomic islets (6). In fact, certain TA system types tend to occur in specific classes of MGEs, supporting the hypothesis that TA systems may have originated multiple times, each primarily associated with distinct mobile elements (3).

*Helicobacter pylori* is a bacterium that colonizes the gastric mucosa of approximately 50% of the global population, establishing typically asymptomatic, chronic infections (7). This infection is often acquired early in life and, in the absence of antibiotic eradication, usually persists for the lifetime of the host. Chronic infection with *H. pylori* is the principal cause of duodenal ulcer disease and classified as a type I carcinogen due to its well-established role in the development of gastric cancer. Its virulence arsenal includes the vacuolating cytotoxin VacA, the CagA oncoprotein, which is delivered into host cells via a type IV secretion system, and various adhesins, flagella, and immunomodulatory enzymes (8). Moreover, its persistence is further aided by the generation of heterogeneous populations through high mutation rates, recombination, and phase variation, as well as immune evasion strategies (9). However, this remarkable ability to survive in the acidic and immunologically hostile gastric environment for decades while evading clearance raises fundamental questions about the adaptive and molecular mechanisms that sustain such a stable, long-term relationship with its host.

*H. pylori* encodes multiple TA systems, including type I AapA/IsoA modules and five type II protein–protein TA systems, implicated in stress adaptation and persistence (10). TA systems are part of a plethora of mechanisms developed by *H. pylori* to chronically survive and multiply in the human stomach niche. Multiple small non-coding RNAs (sRNAs), as well as other mRNA structures, have been described as putative regulators that could be part of TA systems (11), but as of today, only eight have been experimentally validated as part of the *H. pylori* 26695 genome (12). In this review, we aim to describe all the experimentally validated TA systems found in *H. pylori*, along with putative systems predicted through bioinformatic analyses, and discuss their relevance for survival, persister cell formation, and resilience to antibiotic treatment.

## EXPERIMENTALLY VALIDATED TA SYSTEMS IN *H. PYLORI*

To follow up on *H. pylori* TA nomenclature, it is important to notice that HPXXXX refers to American Type Culture Collection (ATCC) strain 700392/26695 locus tags, as it is the reference genome for this bacterium. HPP12_XXXX stands for *H. pylori* P12 chromosome locus tag. Finally, TAXXXX refers to an internal reference code in the TADB database (https://bioinfo-mml.sjtu.edu.cn/TADB3/) (12), with most of these being antisense RNAs without a specific locus tag.

## TYPE I TA SYSTEMS

Type I antitoxins are small RNAs that silence the transcript of their cognate toxins by base pairing of the antitoxin sRNA with the stable mRNA encoding the toxin (13). Although Type I TA systems have been less frequently reported in *H. pylori*, several of them have been experimentally validated.

### TA010085 (*AapaA–IsoA1*)

TA010085 is a type I TA system discovered by Arnion et al., who studied a family of six *cis*-encoded sRNAs with high expression in *H. pylori* strain 26695 (IsoA1–IsoA6). These antisense RNAs were expressed on the opposite strand of the antisense-associated peptide family A (AapA) and have inhibitory capabilities of their cognate sequence. Validation of the system was performed using multiple plasmids carrying different isoforms of *aapA1*/IsoA1. They demonstrated that the toxicity of the AapA peptide without the presence of IsoA1 affects both cell viability (10^4^-fold decrease) and growth rate (immediate growth arrest). Consequently, expression of IsoA1 sRNA restored cell viability by binding to a truncated transcript of *AapA1* full-length mRNA and creating an extended duplex that prevents ribosome binding. The truncation effect requires a 5′−3′ long-distance interaction (LDI), which occurs when both ends of the mRNA are engaged, thereby preventing ribosome interaction with the Shine-Dalgarno sequence and protein synthesis. To restore toxin translation, a 3′-end ribonucleolytic event, termed the ‘mRNA activation step,’ is necessary to remove the LDI. This system has been reported to be present not only on the chromosome but also in MGEs, such as plasmids, prophages, and integrative conjugative elements (ICEs) from other bacteria (11, 14). Notably, in *H. pylori*, this system has been associated with the transition from spiral to coccoid shape, a mechanism associated with persistence that is suggested to enable a state of dormancy (15, 16).

### TA010113 (*AAPA3/ISOA3*)

In 2019, Masachis et al. described a second member of the aapA/IsoA family named aapA3/isoA3, a type I TA system that works by occlusion of the ribosome binding site (RBS) via an anti-Shine-Dalgarno (aSD) sequence located not immediately downstream of the toxin gene but a LDI similar to the one described for TA010085. One third of the suppressors analyzed mapped to non-coding regions of the toxin mRNA, a particular one located at the Shine-Dalgarno (SD) sequence, inhibits translation by stabilizing a hairpin that helps an aSD mask the SD binding sequence (14).

### Other type I TA systems in *H. pylori*

In 2010, Sharma et al. published the primary transcriptome of *H. pylori* strain 26695 under multiple conditions (mid-logarithmic phase, acid stress, contact with epithelial cells, non-responsive liver cells, and cell culture medium alone). Thus, 1,907 transcriptional start sites (TSS) and 337 operons were identified. Most importantly to this review, 27% of the primary TSS were also antisense TSS across the entire genome. Within those, six structurally related sRNA expressed antisense to small open reading frames (ORFs) of homologous 22–30 amino acid peptides (IsoA1-6) and (aapA1-6). They also identified three pairs of antisense RNA-associated peptides (aapC1/2 and aapD) similar to those described in TA systems in *Escherichia coli*, which are known to spread via horizontal gene transfer (17). Both Aap1–IsoA1 and Aap3–IsoA3 have been better characterized, as previously discussed; however, the Aap2–IsoA2 (HP1176), Aap4–IsoA4 (HP1533), Aap5–IsoA5 (HP0024), and Aap6–IsoA6 (HP1515) pairs remain to be further studied.

## TYPE II TA SYSTEMS

In type II TA systems, both the toxin and antitoxin are proteins. The antitoxin exerts its neutralizing function through direct protein–protein interaction with its cognate toxin. These systems are generally organized as operons that are negatively autoregulated at the transcriptional level by either the antitoxin alone or the toxin–antitoxin complex (17–19).

### HP0894–HP0895

The first experimentally validated TA system in *H. pylori* was HP0894–HP0895. Using structural genomics, Han et al. identified similarities between the hypothetical protein HP0894 and known ribonucleases, such as thearchaeal RelE from *Pyrococcus horikoshii* and YoeB from *E. coli*. HP0894 functions as a ribosome-dependent endoribonuclease, becoming active only in the presence of the ribosome or a stimulatory factor that enhances the ribosome’s intrinsic endonuclease activity (20). To confirm the TA nature of HP0894–HP0895, the authors demonstrated the formation of a stable complex, a reduction in cell viability upon HP0894 expression alone, and the inhibition of RNase activity by HP0895 *in vitro*. The ribosome binding region of HP0894 involves three residue clusters, notably C-terminal Arg80 and His84–Phe88 (21).

### HP0892–HP0893

Following the validation of HP0894–HP0895, Han et al. characterized a structurally similar protein, HP0892, and investigated its function (19). Located upstream of *hp0892*, HP0893 forms a stable complex with its partner. Expression of HP0892 alone leads to growth inhibition, an effect reversed by the presence of HP0893. Like HP0894, HP0892 functions as an RNase, with Glu58, Arg82, and His86 identified as key catalytic residues. Interestingly, this TA pair forms heterohexameric complexes and binds its promoter region in a distinct autoregulatory mode compared to other TA systems (19). Mondragon also described that *hp0893–hp0892* was the only type II TA system with boosted expression by acidic pH (22).

### HP0315–HP0316

Unlike the previous TA systems, the HP0315–HP0316 pair is more closely related to the VapD family and shows sequence similarity to the CRISPR-associated protein Cas2 SSO1404. HP0315 exhibits ribonuclease activity specific to single-stranded RNA substrates. However, HP0316 does not bind HP0315 nor display antitoxin activity typical of type II TA systems. Another specific trait of this pair is the operon arrangement, contrary to most VapD proteins from other organisms. As a result, this pair does not follow the canonical TA dynamics associated with growth regulation. Because of its similarity with HP0895–HP0894 (an *H. pylori* bicistronic operon TA system), it was postulated to function as a type II TA system, but a His-tag pull-down assay showed that HP0316 does not bind to HP0315. Nonetheless, the authors propose that HP0315–HP0316 represents a structural intermediate between Cas2 proteins and TA systems, possibly reflecting an evolutionary transition (23).

### HP0967–HP0968

Cárdenas et al. identified HP0967–HP0968 as another VapD-like TA system. HP0967 acts as an RNase, while HP0968 functions as a canonical antitoxin—both neutralizing the toxin and binding to the operon’s regulatory region. This study was the first to analyze TA system expression under multiple stress conditions in *H. pylori*. The *hp0968–hp0967* operon was repressed by metals, such as nickel and iron, moderately upregulated by urea, and strongly induced during mature biofilm formation, epithelial cell interaction (e.g., with AGS cells), and antibiotic exposure, such as chloramphenicol and kanamycin. The authors suggest that the high concentration of urea and low concentrations of nickel and iron in the stomach would induce transcription of type II TA systems in preparation for successful colonization and persistent infection (22).

### HPP12_0452- HPP12_0453 (*TFIT–TFIA*)

The most recently characterized type II TA system in *H. pylori* is TfiT–TfiA, a member of the DinJ-YafQ family. Boampong et al. reported this system as part of either plasmids or the Tfs4 integrative and conjugative element (ICE), specifically within clades T4/AT4 and T6/AT6. TfiT exhibits strong RNase-mediated growth inhibition, a hallmark of TA toxins. Growth restoration is achieved through interaction with TfiA, confirming its role as the corresponding antitoxin (24).

## IMPACT OF TA SYSTEMS ON *H. PYLORI* ADAPTABILITY

TA systems play a pivotal role in mediating bacterial resistance and the formation of persistent populations (25). By exerting selective pressure, these systems facilitate a metabolic shift toward a dormant, drug-resistant state (25, 26). Within *H. pylori*, TA systems are implicated in diverse physiological processes, including morphological transformation, growth arrest, biofilm formation, and antibiotic stress responses. Notably, the transition from spiral-shaped to non-culturable coccoid cells—a process driven by growth arrest—represents one of the most characterized functions of these systems.

El Mortaji et al. (10, 15) demonstrated that the toxin AapA, part of the type I (*AapA-IsoA1*) TA system, targets the *H. pylori* inner membrane without disrupting it and alters peptidoglycan composition, ultimately leading to the coccoid dormant-state phenotype. This transition can be triggered by exposure to several antibiotics and the associated generation of oxidative stress, which subsequently represses the antitoxin promoter. Although there is ongoing debate regarding the relevance of this dormant state for bacterial transmission and antibiotic tolerance, accumulating evidence suggests that coccoid forms remain metabolically active and are key drivers of recurrent infections (27). These cells have been shown to express virulence factors (28), bind to host cells and induce cellular changes (29), and exhibit reduced activation of NF-κB, which may facilitate immune evasion (30). In addition, coccoid forms have been directly observed in human gastric biopsies (31). Collectively, these findings support the idea that coccoid cells represent persister-like forms within heterogeneous *H. pylori* populations, with the capacity to survive antibiotic treatment and potentially revert to a culturable state.

**Fig 1:**
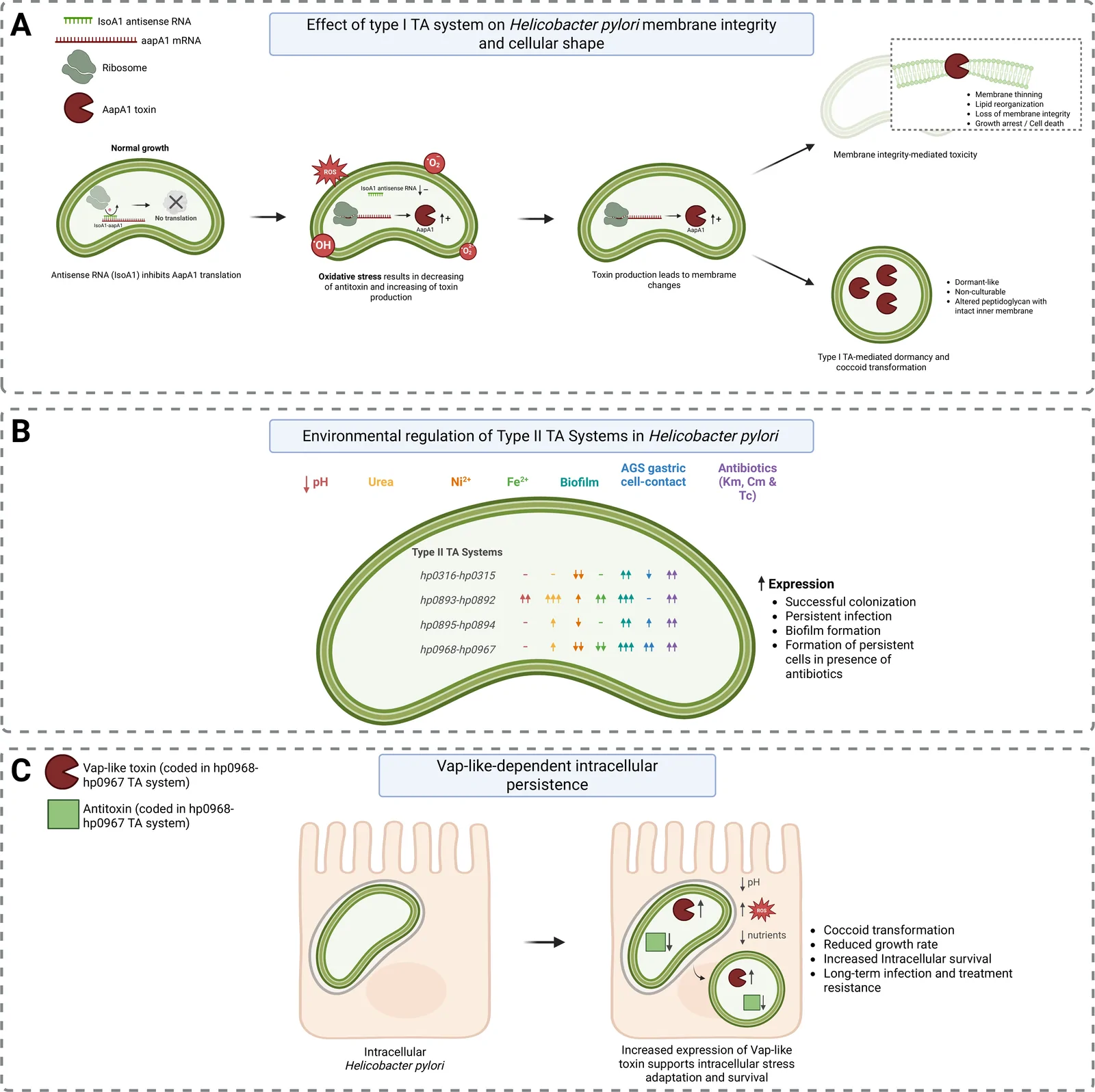
TA-mediated mechanisms contributing to *H. pylori* adaptability. (**A**) Effect of the type I TA system on *H. pylori* membrane integrity and cellular morphology. Increased expression and production of the AapA1 toxin lead to membrane alterations, resulting in toxicity through disruption of membrane integrity and induction of a dormant state via coccoid transformation. (**B**) Environmental regulation of type II TA systems in *H. pylori*. Differential expression of type II TA modules may influence colonization, persistence, biofilm formation, and resilience to antibiotic stress. (**C**) Putative mechanisms of the Vap-like toxin from the type II HP0968–HP097 system based on previously reported activity of the VapD protein (HP0315–HP0316 TA system). Vap-like proteins are expressed, even during the coccoid dormant state, and may enhance intracellular survival, long-term infection, and treatment resistance.

Nonetheless, toxin AapA1 has also been reported in other studies (11, 32) to interact with and destabilize the bacterial membrane, specifically through its basic C-terminal domain, thereby generating a toxic effect (Fig. 1A). These distinct effects on bacterial biology mediated by the same TA-derived toxin underscore the need to further explore the role of this and other potential TA-mediated toxins in the biology of *H. pylori* and other clinically relevant bacteria. This is particularly important given that the modulation of such systems could provide alternative therapeutic strategies to conventional antibiotics (27), for example, by enhancing the intrinsic toxic activity against bacterial cells and disrupting the formation of dormant cells. A similar approach was evaluated by Martin and colleagues (33) who reported the design and synthesis of novel RNA binders that inhibit RNA–RNA interactions in type I systems, thereby activating toxin production and promoting bacterial cell death.

While the general pathogenesis of *H. pylori* is well documented, the regulatory role of TA systems represents a nascent field of inquiry. Research by Cardenas-Mondragón et al. (22) established that certain type II TA systems are upregulated in response to environmental stressors, such as fluctuations in urea, nickel, and iron concentrations. Their findings further indicate that TA expression is modulated during biofilm formation and interactions with AGS cells. Additionally, exposure to antibiotics like kanamycin and chloramphenicol disrupts the toxin–antitoxin equilibrium, suggesting that these systems enhance bacterial resilience to antibiotic treatment and promote persistence within the hostile gastric niche by inducing a dormant state (22, 34, 35) (Fig. 1B). Furthermore, the gene *cptA* reported as a potential TA-associated gene in *H. pylori* (36) has been characterized as part of type IV TA systems in *Acinetobacter baumannii* and is potentially implicated in antibiotic and stress responses (37). These findings suggest the existence of additional genes that may contribute to pathogen adaptability.

Even though *H. pylori* is not a classical intracellular pathogen, intracellular survival has been described as a novel aspect of its pathogenicity, allowing persistence as it enables *H. pylori* to evade the immune system (38). Some classical virulence factors include VacA (39) and the capacity to tamper with autophagic flux in epithelial cells (40). In this context, VapD (HP0315) protein has been identified as an important component for intracellular survival in *H. pylori* and is expressed, even in the non-culturable coccoid state; this gene is specifically induced upon contact with gastric epithelial cells, driving the transition into a viable but non-culturable (VBNC) state (41). In the HP0968–HP097 TA system of *H. pylori*, the toxin component (HP0968) is classified as a Vap-like protein belonging to the same family as the VapD toxin (22). This observation reinforces the hypothesis that HP0315–HP0316 may represent an evolutionary transition between Cas2-like proteins and TA systems, as previously mentioned (23). Moreover, this classification suggests that the HP0968–HP097 type II TA system could contribute to *H. pylori* intracellular survival, long-term infection, and resilience to antibiotic treatment through mechanisms similar to those described for VapD (41) (Fig. 1C). However, this remains speculative, as there is currently no experimental evidence demonstrating whether the Vap-like protein of the HP0968–HP097 system exerts effects similar to those of the previously characterized VapD protein (HP0315) in the same pathogen.

## POTENTIAL UNEXPLORED TA SYSTEMS IN *H. PYLORI*

The increasing availability of sequenced genomes in public repositories has driven the development of several bioinformatic tools to analyze these large data sets and confer evolutionary and putative functional information of organisms. In 2022, Gupta et al. (36) investigated the genome of the *H. pylori* strain 26695 using tools, such as TASmania (42), TADB 2.0 (43), and SLING (44), to identify TA systems based on both characterized and putative components of type I to IV TA loci. Their analysis revealed 80 putative TA genes in *H. pylori*, highlighting the high potential prevalence of TA systems in this human pathogen. Among these, 12 shared homology with experimentally validated TA systems in *E. coli*, five in *Mycobacterium tuberculosis*, and three in *Vibrio cholerae*, demonstrating the utility of bioinformatics in uncovering novel TA candidates in *H. pylori* and other pathogens.

More recently, the TADB database was updated to version 3.0 in 2024 (12), incorporating data from 34,789 prokaryotic genomes and linking TA systems to MGEs. According to the web server, *H. pylori* is one of the bacterial species with the highest number of experimentally validated TA systems, with eight found in strain 26695 and one in strain P12. Nevertheless, the database bioinformatically predicts a total of 611 TA systems across *H. pylori* strains, with an average of 1.7 TA systems per strain. Although this represents a relatively low number of potential TA systems compared with other pathogens, such as *E. coli* (59,175; 21.2 per strain), *Klebsiella pneumoniae* (22,173; 14 per strain), or *M. tuberculosis* (21,043; 59.1 per strain), it is still comparable to others, such as *Haemophilus influenzae* (654; seven per strain) and *Proteus mirabilis* (613; 5.8 per strain) (12). This underscores the significant number of uncharacterized TA systems in this organism, which may contribute to its biology.

In addition, Mendoza et al. (45) analyzed all plasmid sequences associated with *H. pylori* available in several public databases and identified a potential TA system based on plasmid-encoded filamentation induced by cAMP (Fic) proteins. Fic proteins are well-known bacterial effectors with toxic activity and have been linked to TA systems type II in *Bartonella* spp., *Campylobacter fetus* subsp. *venerealis* (46), and other bacterial species (47). The authors found that several abundant plasmid sequences carried two Fic proteins arranged in tandem. Phylogenetic analysis and amino acid motif characterization suggested that one of the Fic proteins may function as an antitoxin, as it contained a conserved suppression motif capable of inhibiting the activity of the toxin Fic protein. A similar mechanism has been previously reported and experimentally validated in *C. fetus* subsp. *venerealis* (46), a phylogenetically close relative of *H. pylori*, supporting the plausibility of a plasmid-mediated TA system in *H. pylori*. These putative systems warrant further investigation, particularly through experimental approaches, to elucidate their roles in *H. pylori* stress adaptation, persistence, and pathogenicity.

## CONCLUSION

TA systems in *H. pylori* have likely emerged as key players in stress adaptation, morphological transformation, dormancy induction, biofilm formation, and antibiotic tolerance. Experimentally validated type I and II systems demonstrate that these modules are actively involved in growth regulation and in the transition to coccoid or viable but non-culturable states, processes potentially linked to chronic infection and treatment failure. Moreover, the association of some putative TA systems with MGEs suggests an evolutionary interplay between genome plasticity and bacterial fitness in *H. pylori*. Despite advances in functional characterization, the large number of predicted but unvalidated TA systems highlights substantial gaps in our understanding of their biological roles. Future studies integrating molecular, structural, and *in vivo* approaches are necessary to clarify their contribution to adaptability, virulence regulation, and antibiotic resistance. Elucidating the full repertoire and functional diversity of TA systems in *H. pylori* may uncover novel targets for therapeutic intervention aimed at disrupting bacterial persistence and improving eradication strategies.
